# Barriers and Facilitators to Being Physically Active on a Rural U.S. Northern Plains American Indian Reservation

**DOI:** 10.3390/ijerph111112053

**Published:** 2014-11-21

**Authors:** Lisa Jahns, Leander R. McDonald, Ann Wadsworth, Charles Morin, Yan Liu

**Affiliations:** 1 Grand Forks Human Nutrition Research Center, Agricultural Research Service, United States Department of Agriculture, 2420 2nd Avenue North, Grand Forks, ND 58203, USA; 2 Spirit Lake Dakota Nation, P.O. Box 359, Fort Totten, ND 58335, USA; E-Mail: lrmcdonald@spiritlakenation.com; 3 Cankdeska Cikana Community College, Spirit Lake Dakota Nation, P.O. Box 269, Fort Totten, ND 58370, USA; E-Mail: ann.wadsworth@littlehoop.edu; 4 Tate Topa Tribal School, Spirit Lake Dakota Nation, P.O. Box 211, St. Michael, ND 58370, USA; E-Mail: cmorin@gondtc.com; 5 Department of Pediatrics, Children’s Nutrition Research Center, Baylor College of Medicine, 1100 Bates Street, Houston, TX 77030, USA; E-Mail: yliu3@bcm.edu

**Keywords:** American Indians, physical activity, rural, reservation, barriers, facilitators

## Abstract

The objective of the present study was to identify barriers to and facilitators of physical activity among American Indian adults living on a rural, U.S. Northern Plains reservation using the nominal group technique (NGT). NGT is a method of data generation and interpretation that combines aspects of qualitative (free generation of responses) and quantitative (systematic ranking of responses) methodologies. Adults participated in one of two NGT sessions asking about either barriers to (n = 6), or facilitators of (n = 5), being physically active. Participants nominated and ranked 21 barriers and 18 facilitators. Barriers indicated lack of knowledge of how to fit physical activity into a daily schedule, work, caring for family members, and prioritizing sedentary pursuits. Other responses included environmental barriers such as lack of access and transportation to a gym, unsafe walking conditions, and inclement weather. Facilitators to following recommendations included knowledge of health benefits of physical activity and the perception of physical activity as enjoyable, including feeling good when working out. Environmental facilitators included being outdoors walking and biking as well as parks and exercise facilities. Responses provided direction for locally designed community-based programs to promote facilitators and decrease barriers to individual’s engagement in physical activity.

## 1. Introduction

Nearly 40% of American Indian adults are obese [1] and American Indians have the highest prevalence of diabetes in the United States [2]. The rates of other chronic diseases are also very high in this population [3,4]. Regular physical activity is associated with maintenance of a healthy body weight and decreased risk of some chronic diseases [5,6,7], yet most American Indian adults get either inadequate or no regular activity [1,8,9,10,11,12].

A recent systematic review of physical activity levels of Native Americans in the U.S. and Canada synthesized results from 89 studies [12]. They found that based upon self-reported activity, 27.2% (95% CI 26.9–27.5) of individuals met recommendations, 22.1% (95% CI 21.8–22.4) had inadequate activity, and 47.9% (95% CI 47.6–48.2) were not active. However, when objective measurements such as pedometers and accelerometers were used, only 9% of individuals met recommendations [12].

The obesity rate among Northern Plains American Indians is 34% [13]. Approximately 30% of American Indian adults living in the Northern Plains do not participate in any leisure-time physical activity [14] and 30% report being physically inactive [13]. The little research of factors associated with physical activity has for the most part been conducted by designers of physical activity interventions [15,16]. Most interventions have targeted children and were conducted in the southwest U.S. [17]. Surprisingly little information on modifiable factors affecting adults’ physical activity is found in the literature, particularly in the Northern Plains area. To our knowledge, only one article has focused on barriers to being physically active reported by Northern Plains American Indians, and that only in women. Barriers included both personal reasons (lack of time, lack of willpower) and environmental obstacles such as unsafe roads and fear of animals outside [18]. Rural residence itself may be an independent risk factor for obesity and inadequate levels of activity. Rural residents have been reported to be less active than their urban counterparts [19] or to engage in less high-intensity activity [20]. However, studies have also reported that urban residents are less active than those in small rural communities, and that there are no urban-rural differences in physical activity in the Midwest [21]. Therefore, the objective of the present study was to identify barriers to and facilitators of physical activity among American Indian adults living on a rural, Northern Plains reservation. This information will help to inform the design of local interventions to promote PA in smaller, rural tribal communities, particularly in the Northern Plains region of the U.S..

## 2. Methods

The present study was conducted as an adjunct to the Healthy Eating and Lifestyle for Total Health (HEALTH) study, a larger national study of African-, European-, and Hispanic American adults and fifth-grade children. The focus of both studies were barriers and facilitators to following the 2005 Dietary Guidelines for Americans as portrayed by MyPyramid, a set of diet and physical activity recommendations displayed as a consumer-friendly educational icon. Details may be found elsewhere [22,23].

### 2.1. Study Subjects

Participants took part in either a barriers (n = 6) or a facilitators (n = 5) Nominal Group Technique (NGT) session. Participants were enrolled tribal members who self-identified as American Indian and were primary caregivers of a fifth-grade child. Recruitment methods included flyers placed in reservation schools and the local boys and girls club, and word-of-mouth. Participants completed a demographic form before sessions were conducted at a local school on the reservation. Sessions lasted approximately one hour and participants received a $50 gift card. The study was approved by the University of North Dakota Institutional Review Board (No. 200906-376) and by tribal resolution and all participants provided written consent.

### 2.2. Nominal Group Technique

NGT is a method of data generation and interpretation that combines aspects of qualitative (free generation of responses) and quantitative (systematic ranking of responses) methodologies [24]. In focus groups, individuals are encouraged to interact with each other and to generate as many ideas as possible. The NGT is also an idea-generating approach, but is highly structured and results in easily interpretable ordinal data that reflects the priorities of both individuals and the group [25,26,27].

### 2.3. Data Collection and Analysis

Participants sat in a circle. The meetings were conducted on the reservation by two community members who were trained to be group facilitators. One facilitator acted as a moderator or leader and the other recorded verbatim responses on a flip-chart.

Most Americans do not know what the physical activity recommendations are, therefore a short presentation was shown on a laptop and participants received handouts outlining the recommendations. This presentation and handout defined physical activity, gave examples, and showed the recommendations. In step one, participants worked silently using a provided worksheet and wrote brief responses to a single question “what sorts of things make it hard (barriers group); or easier (facilitators group) for *people* to follow the MyPyramid recommendations for physical activity?”. It is important to note that individuals were asked to consider the question from a third-person point of view, in this case, for people in general. Doing so minimizes potential intimidation of sharing personal beliefs in front of the group.

In step two, participants, one at a time, read aloud one idea from their worksheets until all exhausted their list. Responses were numbered and recorded on a flip-chart. In step three, the facilitator posted all responses on the wall and led participants through a clarification period to ensure understanding of all responses and asked if there was anything else to add to the list. Next, participants were given five index cards and asked to write the five responses, along with their number, from the chart that were most important to them *personally*; one on each card. Participants then individually ranked, or voted on, the responses by choosing the most important response and writing a number five on the index card. The least important response received a rank (vote) of one. Steps were repeated with votes two (second most important) and four (second least important); the final response was assigned a vote of three. Ranked votes for responses were summed and numerically represent the group’s priorities. In tables one and two, votes in the second column (voting) were taken from the index cards; for example, under the barrier response “jobs”, five out of the six participants individually chose that response as most salient to themselves. Four people gave it a rank of five and one person gave it a rank of four, so the sum of the votes (column three) was 24. Responses nominated by individuals but did not receive votes are also presented. Responses are presented verbatim as recorded on the flip-chart; therefore some responses may be incomplete or have grammatical errors.

## 3. Results

### 3.1. Sample Characteristics

Six adults participated in the barriers session and five participated in the facilitators’ session. Twenty-one barriers and 18 facilitators to being physically active on the reservation were nominated. Of the 11 total participants, two-thirds were men, evenly split between married and never married, and two-thirds were employed. Another two-thirds lived in household comprised of four to six people and almost all lived in a home with at least three children under the age of 18. Seven of the 11 people had attended at least some college or technical school.

### 3.2. Summary of Responses to Barrier Question

Participants voted for 12 of the nominated barriers (Table 1). The core barriers ranked by adults can be grouped into three general themes: (1) obligations and time management (42%; jobs, time, and family); (2) personal (25%; being on computer, watching TV, being tired); and (3) environmental (33%; inclement weather, unsafe walking conditions). Barriers related to obligations and time management included the following: “jobs”; “time”; “taking care of children all the time”; “family functions”; and “single parent”. Personal barriers included “being on computer all day”; tired after working cooking for kids and get tired after eating”; and “watch too much TV a lot of movies”. Environmental barriers were “no access to gym”; “there are places to go walking but because of dogs may attack”; “transportation”; and “long winter months can’t do any walking with all the snow”.

### 3.3. Summary of Responses to Facilitator Question

Adults voted for 15 of the nominated facilitators (Table 2). The core facilitators ranked by adults can be grouped into two general themes: (1) environmental (80%; being outdoors, having equipment and environmental resources) and (2) internal (20%; feeling good, knowing the benefits).

Environmental facilitators included the following: “outdoors”; “going walking with my kids”; “bike path is available”; “casino swimming pool”; “rec. is always open”; “having a bike”; “parks”; “Sully hill is always available”; “good pair of shoes”; “hand weights”; “Anytime fitness is always open”; and “working”. Internal facilitators included “feel good when I work out”; “I have to for my heart”; and “I know it’s good for me”.

**Table 1:** Barriers to meeting physical activity recommendations.

Barrier Responses	Voting	Sum of Votes
Jobs	4, 5, 5, 5, 5	24
Time	2, 5, 5, 2, 4	18
No access to gym	4, 4, 3	11
Being on computer all day	3, 3	6
Taking care of children all the time	3, 2	5
Family functions	2, 3	5
There are places to go walking but because of dogs may attack	4	4
Single parent	4	4
Tired after working coking for kids and get tired after eating	3, 1	4
Watch to much tv a lot of movies	1, 1, 2	4
Transportation	2, 1	3
Long winter months can’t do any walking with all the snow	1	1
Cost	1	1
Education		0
Appointments		0
People smoking too much		0
Sickness		0
Watching regular programs on TV		0
Washing clothes, daily duties, can’t do what you want to do		0
Looking forward sporting events on TV		0
Both diabetic don’t know how much exercise to get don’t want to over do it		0

**Table 2:** Facilitators to meeting physical activity recommendations.

Facilitator Responses	Voting	Sum of Votes
Outdoors	5, 4	9
Going walking with my kids	4, 5	9
Feel good when I workout	4, 5	9
Bike path is available	3, 5, 1	9
I have to for my heart	2, 4	6
Casino swimming pool	5	5
Rec. is always open	2, 3	5
Having a bike	4	4
I know it’s good for me	3, 1	4
Parks	3, 1	4
Sully Hill is always available	2	4
Good pair of shoes	3	3
Hand weights	2	2
Anytime fitness is always open	1	1
Working	1	1
I enjoy softball and going to my kid’s games		0
Lower my diabetes		0
Casino fitness room		0

## 4. Discussion

This study describes American Indians’ barriers and facilitators to being physically active while living on a rural U.S. Northern Plains reservation. Both groups discussed the environment in different contexts. Although environmental facilitators involved enjoyable outside activities, external barriers included perceived danger from dogs and long, extreme winters, making being outside difficult. As the average winter temperature in this area is around −18 ºC and often reaches −30 ºC and below, public health interventions and community infrastructure development should try to include areas for people to be physically active, especially indoor areas during inclement weather. At the person level, obligation and time management barriers suggest that culturally-appropriate community programs to increase awareness of the health and well-being effects of regular physical activity and decrease preferences for sedentary activities should also include time management strategies. Several barriers identified were strikingly similar to those reported in a Northern Plains tribal community 15 years ago [18]. Harnack *et al*. reported that lack of child care, lack of time, and safety concerns (such as snakes and stray dogs) were the most frequently-mentioned barriers to physical activity among Northern Plains women [18]. Social support and cultural identity have also been identified with physical activity level, with the highest levels of activity found among adults who speak both English and traditional language compared to either alone [28,29]. Similar to this study, barriers presented by the physical environment have been identified in qualitative studies of women [18,30] but a review of the correlates of physical activity in Native Americans concluded that while age, gender, and social support are important factors to consider, there is no empirical evidence to suggest that the physical environment is a barrier [29]. However, the authors concluded that the literature is sparse and more research is needed to inform interventions. Much of the research describing barriers and facilitators to being physically active has been conducted in groups targeted by demographic characteristics or geographic location, limiting the ability, like these results, to generalize to other populations. Some barriers and facilitators identified in this study are similar to other studies in the Midwestern U.S. Environmental barriers such as bad weather and lack of facilities were identified by rural adults [31,32] and Somali men [33]. African American women also cited lack of time and motivation, long work hours and hard manual labor, in addition to lack of safe places to be active. Individual facilitators included positive health benefits and personal enjoyment. Environmental facilitators were also similar to those reported in this study [34]. In fact, results of this study suggest that while interventions need to be tailored to the specific community, many of the barriers and facilitators are similar to those experienced by non-reservation individuals. For instance, social support and community are often cited as important factors for changing health behaviors in American Indian communities, but this group identified factors related to lack of time, which is cited by many different groups, as the most relevant barriers to being physically active. This indicates that while environmental changes are desired by participants, individual—level factors must be addressed concurrently or even beforehand to promote health-related behavior change.

### Limitations and Strengths

Limitations of this study include the small sample size and use of a single group for each question. However, NGT has previously been used with single groups [35] and with small samples [26,35] as response saturation with this method is reached when participants no longer provide new answers. The NGT method does not allow for exploration of the responses identified in sessions. Participant responses may be as brief or detailed as desired, and as long as the group indicates understanding of the responses, there is no additional clarification. Its strength lies in the ordinal data output—the ranked responses representing the most important factors identified by the group. This information may be used for further formative research such as development of questions for focus groups to explore the ranked factors in more detail. The ranked NGT responses may also be used in quantitative methods, to create questionnaires that may be disseminated to the community. Further research is needed to delineate and expand upon many of the responses such as “jobs”. Some responses, such as threat from dogs and extreme weather, may not require much more clarification. The sample was self-selected with a large number of men and over half of participants had attended at least some college, therefore participants may not be reflective of the general reservation population. Recruitment on rural reservations is difficult [36], even with the provision of monetary incentives and snacks and this study was no exception. Lack of transportation was a barrier to participation, as were winter storms, flooding, school schedules, and district and regional tournaments. As American Indian tribal communities across the U.S. vary by geography, culture, and economies, this study is not generalizable to other reservations, but is informative for other Northern Plains communities.

Although small, this study makes a contribution to the literature by describing elements that make it harder or easier to be physically active for an understudied population at high risk of inactivity and obesity. For instance, it is of interest that some of the environmental items described, such as feral dogs constituting a safety hazard, have been mentioned in the literature before and may be unique to rural reservations. Removing the threat of dogs may be a lesser expense than other infrastructure changes and could help to promote walking. The results provide direction for locally designed community-based programs to promote facilitators and decrease barriers to individual’s engagement in physical activity.

While not part of the stated research objectives, other benefits emerged from this project. The NGT is versatile and has been used for formative research in a variety of health areas [26,27,35]. Researchers from this community learned to implement and interpret research questions using the NGT method, and will partner with the tribal council to develop strategic plans for the reservation. The results of this study were disseminated among the tribe and have already been translated into practice by use in local grant proposals to improve recreational infrastructure and health promotion programs.

## 5. Conclusions

This research describes environmental and individual-level barriers and facilitators to being physically active reported by American Indian tribal members living on a rural reservation. Results suggest that the environment can either act as a barrier to or facilitator of physical activity, although internal factors such as time management skills and motivation to replace television or other sedentary activities are key intervention points to effect healthy behavior change.
